# Blueberry and/or Banana Consumption Mitigate Arachidonic, Cytochrome P450 Oxylipin Generation During Recovery From 75-Km Cycling: A Randomized Trial

**DOI:** 10.3389/fnut.2020.00121

**Published:** 2020-08-07

**Authors:** David C. Nieman, Nicholas D. Gillitt, Guan-Yuan Chen, Qibin Zhang, Wei Sha, Colin D. Kay, Preeti Chandra, Kristine L. Kay, Mary Ann Lila

**Affiliations:** ^1^Human Performance Laboratory, Appalachian State University, North Carolina Research Campus, Kannapolis, NC, United States; ^2^David H. Murdock Research Institute, Kannapolis, NC, United States; ^3^UNCG Center for Translational Biomedical Research, University of North Carolina at Greensboro, North Carolina Research Campus, Kannapolis, NC, United States; ^4^Bioinformatics Services Division, University of North Carolina at Charlotte, North Carolina Research Campus, Kannapolis, NC, United States; ^5^Food Bioprocessing and Nutrition Sciences Department, Plants for Human Health Institute, North Carolina State University, North Carolina Research Campus, Kannapolis, NC, United States; ^6^Department of Nutrition, University of North Carolina at Chapel Hill, Nutrition Research Institute, Kannapolis, NC, United States

**Keywords:** exercise, lipid mediators, carbohydrate, polyphenols, metabolites, inflammation, fatty acids

## Abstract

Oxylipins are bioactive lipid oxidation products, have vital regulatory roles in numerous physiological processes including inflammation, and can be impacted by diet. This study determined if 2-weeks of blueberry and/or acute banana ingestion influenced generation of n-6 and n-3 PUFA-derived oxylipins during recovery from exercise-induced physiological stress. Cyclists (*n* = 59, 39 ± 2 years of age) were randomized to freeze-dried blueberry or placebo groups, and ingested 26 grams/d (1 cup/d blueberries equivalent) for 2 weeks. Cyclists reported to the lab in an overnight fasted state and engaged in a 75-km cycling time trial (185.5 ± 5.2 min). Cyclists from each group (blueberry, placebo) were further randomized to ingestion of a water-only control or water with a carbohydrate source (Cavendish bananas, 0.2 g/kg carbohydrate every 15 min) during exercise. Blood samples were collected pre- and post-2-weeks blueberry supplementation, and 0, 1.5, 3, 5, 24, and 48 h-post-exercise. Plasma oxylipins and blueberry and banana metabolites were measured with UPLC–tandem MS/MS. Significant time by treatment effects (eight time points, four groups) were found for 24 blueberry- and seven banana-derived phenolic metabolites in plasma (FDR adjusted *p* < 0.05). Significant post-exercise increases were observed for 64 of 67 identified plasma oxylipins. When oxylipins were grouped relative to fatty acid substrate [arachidonic acid (ARA), eicosapentaenoic acid (EPA), docosahexaenoic acid (DHA), α-linolenic acid (ALA), linoleic acid (LA)], and enzyme systems [cytochrome P450 (CYP), lipoxygenase (LOX)], banana and blueberry ingestion were independently associated with significant post-exercise reductions in pro-inflammatory ARA-CYP hydroxy- and dihydroxy-eicosatetraenoic acids (HETEs, DiHETrEs) (treatment effects, FDR adjusted *p* < 0.05). These trial differences were especially apparent within the first 3 h of recovery. In summary, heavy exertion evoked a transient but robust increase in plasma levels of oxylipins in cyclists, with a strong attenuation effect linked to both chronic blueberry and acute banana intake on pro-inflammatory ARA-CYP oxylipins.

## Introduction

Acute carbohydrate ingestion from a variety of sources including fruits during prolonged and intensive exercise attenuates post-exercise increases in total blood leukocytes, IL-6, IL-10, and other biomarkers for inflammation (1–5).

In a previous study (4, 5), we used global metabolomics to compare metabolite shifts in 20 athletes cycling 75-km while ingesting water alone, a 6% carbohydrate beverage, and two different types of bananas (Cavendish, mini-yellow) using a randomized, crossover design. Acute ingestion of both types of bananas resulted in significant shifts in the plasma metabolome compared to water alone or a 6% sugar beverage, and was associated with shifts in unique banana metabolites. Post-exercise inflammation and plasma levels of cytochrome P450-generated oxylipins were attenuated in the banana and sugar water trials compared to water alone (5). Oxylipins are bioactive oxidation products generated during stressful exercise from the metabolism of n-6 and n-3 polyunsaturated fatty acids (PUFAs) by cyclooxygenase (COX), lipoxygenase (LOX), and cytochrome P450 (CYP) enzyme systems (5). Despite the distinctive increase in plasma banana-related metabolites, the overall rate of recovery and attenuation in post-exercise inflammation and oxylipins did not differ between the various carbohydrate sources (6% sugar beverage and the two types of bananas), suggesting carbohydrate was responsible. This finding was contrary to our original hypothesis linking banana-related metabolites to reduced oxylipin generation and may have been related in part to the low total polyphenol content of bananas (2.55 mg/100 grams) (4, 5).

Blueberries are a highly concentrated source of polyphenols (716 mg/100 grams) (6). In a previous global metabolomics-based study conducted in our laboratories, 2-weeks ingestion of blueberry and green tea polyphenols vs. placebo was associated with significant increases in gut-derived phenolic metabolites (7). Polyphenols such as blueberry anthocyanins undergo extensive biotransformation after ingestion with the majority reaching the lower bowel where microbial degradation produces gut-derived phenolic metabolites that can be reabsorbed into the systemic circulation and exert a variety of bioactive effects (8–11).

Oxylipins regulate numerous physiological processes, and the influence of exercise and diet on their production and activity is an emerging area of investigation (5, 12–15). Hypothesized roles for flavonoids on oxylipin production exist, but human data are lacking (16–21).

We sought to extend the finding from our previous study linking acute carbohydrate ingestion with decreased post-exercise plasma levels of CYP-generated oxylipins (5). The purpose of this study was to investigate the combined and independent influences of 2-weeks blueberry ingestion and acute carbohydrate ingestion from bananas on plasma gut-derived phenolics and post-exercise plasma oxylipin concentrations in trained cyclists using quantitative broad-spectrum metabolomics.

## Materials and Methods

### Research Design

**Figure 1:**
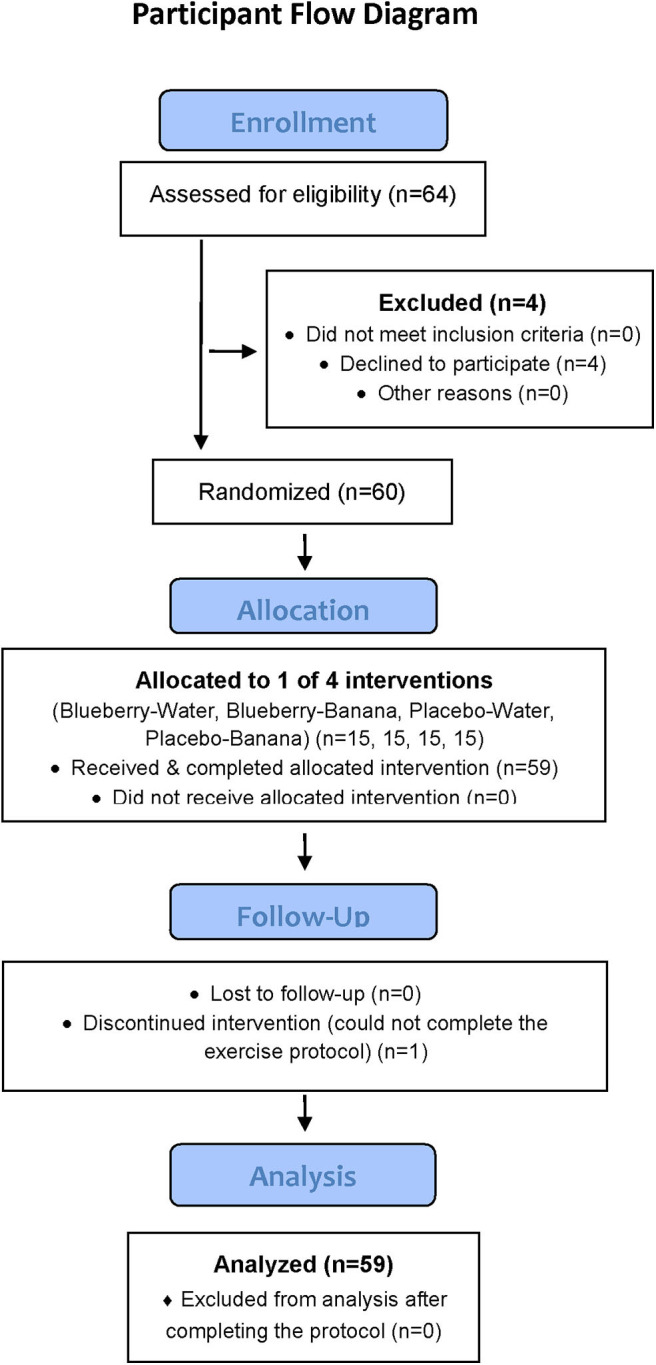
CONSORT participant flow diagram.

This study employed a randomized, double blind, placebo controlled, parallel four group design, with a 2-weeks supplementation period (freeze-dried blueberry powder or placebo) followed by a 75-km cycling time trial (while consuming water only or water with bananas) and 2.5 days of recovery monitoring. Double-blind procedures were used for the blueberry and placebo 2-weeks supplementation period only. Participants were randomized using http://www.randomization.com/ to one of four groups using a 1:1 allocation ratio: blueberry-water (BLUE-WAT), blueberry-banana (BLUE-BAN), placebo-water (PLAC-WAT), and placebo-banana (PLAC-BAN). Blood samples were collected pre- and post-2-weeks supplementation, and then 0, 1.5, 3, 5, 24, and 48 h-post-exercise, with plasma aliquots measured for oxylipins (primary) and gut-derived phenolics (secondary). The Consolidated Standards of Reporting Trials (CONSORT) flow diagram and checklist are available as Figure 1 and Checklist S1.

### Participants

Study participants included healthy, non-smoking male and female cyclists (ages 18–55 years) who regularly competed in road races and were capable of cycling 75-km at race pace in a performance laboratory setting. Of 64 participants screened for entry into the study, 60 met inclusion criteria and consent to join the study, and were randomized into one of four groups, with 59 completing all aspects of the study protocol (Figure 1). During the 2.5-weeks period when data was collected, participants maintained their typical training regimen, and avoided the use of vitamin and mineral supplements, herbs, and medications that influenced inflammation and immune function. Participants signed informed consent and study procedures were approved by the Institutional Review Board at Appalachian State University. The study was conducted during the fall of 2017 at the Human Performance Laboratory, North Carolina Research Campus, Kannapolis, NC. Trial registration: NCT03445234: https://clinicaltrials.gov/ct2/show/NCT03445234.

One-to-two weeks prior to the start of the study, study participants reported to the Human Performance Lab for orientation and baseline fitness testing. Study participants were tested for maximal aerobic capacity (VO_2max_) during a graded, cycle ergometer test with the Cosmed CPET metabolic device (Cosmed, Rome, Italy). Body composition was measured with the Bod Pod body composition analyzer (Life Measurement, Concord, CA). Demographic and training histories were acquired with questionnaires.

### 2-Weeks Supplementation Period

Study participants returned to the performance laboratory after baseline testing and orientation. A blood sample was collected in an overnight fasted state. Study participants were randomized to blueberry and placebo groups, with freeze-dried supplements used for 2-weeks prior to participation in a 75-km cycling time trial. This dosing regimen was chosen based on data from a previous study conducted by our research group demonstrating that metabolites characteristic of gut bacteria metabolism of polyphenols were increased after 2 weeks supplementation (7). The blueberry powder supplement (26 g per day or ~1 cup fresh blueberries equivalent) was provided in 14 vacuum-sealed packets consisting of freeze-dried, lowbush wild blueberries (*Vaccinium angustifolium* Ait) sourced from Allen's Blueberries (Ellsworth, ME) and freeze-dried by Future Ceuticals (Momence, IL). This product contained all of the polyphenols, fibers, and natural sugars found in fresh wild blueberries (6). The lyophilized powder (0.5 g) was blended with 8 mL of acidified 70% aqueous methanol (0.5% acetic acid) for 2 min, centrifuged, extracted, and filtered. Total anthocyanins were determined by HPLC and quantified as mg cyanidin-3-glucoside equivalent (CGE). Total phenolic content was determined with Folin-Ciocalteu reagent according to a microplate-adopted method (21). Concentrations were expressed as gallic acid equivalents (GAE) based on a created gallic acid standard curve. The analysis showed that each 26 mg serving of the blueberry powder provided a total polyphenol content of 1,059 mg GAE, and a total anthocyanin content of 345 mg CGE. The carbohydrate- and fiber-matched placebo powder looked identical and was formulated with these ingredients: blueberry flavoring and aroma (8.9%), coloring (1.05% purple lake, 0.75% red lake, 0.45% blue 2 lake, 0.03% red dye, 0.008% blue 2 dye), glucose (32%), fructose (35.5%), citric acid (0.77%), cellulose (15%), fibersol-2 (1.8%), xanthan gum (1%), pectin (1.09%), and silica (0.99%). Study participants consumed the blueberry and placebo powders by mixing them in water, fruit juices, yogurts, or other products during the first meal of each day for 2 weeks. Compliance (100%) was monitored by reviewing adherence to procedures when the participants returned to the lab after the 2-weeks supplementation period.

### 75-km Cycling Time Trial Session

Procedures for the 75-km cycling time trial session were similar to what we have previously reported in other studies (1, 2, 4, 5). During the 3-days period prior to the 75-km cycling trial, subjects tapered exercise training and ingested a moderate-carbohydrate diet using a food list restricting high fat foods, visible fats (e.g., oils, butter, margarine), and polyphenols. Participants recorded all food and beverage intake during the 3-days period, with macro- and micro-nutrient, and flavonoid intake assessed using the Food Processor dietary analysis software system (Version 11.1, ESHA Research, Salem, OR, USA).

On the cycling intervention day, study participants reported to the performance lab in an overnight fasted state (7:00 am), turned in the 3-days food record, provided a blood sample, ingested water (*ad libitum*), and then cycled 75-km at high intensity while ingesting water alone (3 mL/kg every 15 min) or bananas (adjusted to 0.2 g carbohydrate per kilogram of body weight every 15 min) and water (3 mL/kg every 15 min) (randomized assignment). Bananas were provided by Dole Foods (Westlake Village, CA, USA) at the accepted ripening stage (full yellow, stage six). Blood samples were collected at 0, 1.5, 3, and 5 h-post-exercise, and then again in overnight fasted states the following two mornings at 7:00 am (24 and 48 h-post-exercise).

Participants cycled the 75-km course as fast as possible using their own bicycles on CompuTrainer Pro Model 8001 trainers (RacerMate, Seattle, WA). The CompuTrainer MultiRider software system (version 3.0, RacerMate, Seattle, WA) was used to simulate a moderately difficult, mountainous 75-km course with continuous workload monitoring (watts) and total time recorded. Heart rate and rating of perceived exertion (RPE) were recorded every 30 min. Oxygen consumption and ventilation were measured using the Cosmed Quark CPET metabolic cart after 16 and 55 km cycling (level sections of the course).

Blood samples were taken via venipuncture immediately after completing the 75-km time trial, and then 1.5-h post-exercise. Subjects were allowed to shower and change clothes. Subjects ingested no food or beverage other than water (7 mL/kg) during the 1.5 h post-exercise period. After the 1.5-h recovery blood sample, a standardized meal (low in polyphenols) adjusted to 12 kcal/kg body weight was provided to participants. The lunch included grilled chicken, rice, corn, green beans, salt, and water. Participants randomized to the blueberry group consumed their daily dose (26 g) during the meal. Additional blood samples were taken via venipuncture 3 and 5-h post-exercise, with subjects resting quietly and ingesting water (7 mL/kg per hour). At 3:00 pm, subjects left the lab, with instructions to adhere to the food list requirements, and keep exercise training intensity moderate. Recovery blood samples were collected the following two mornings in an overnight fasted state. The final 26 g blueberry dose was consumed following the 24 h recovery blood sample.

### Serum Glucose and Plasma Cytokines (IL-6, IL-1ra)

Blood samples were collected into vacutainer whole blood collection tubes with EDTA and centrifuged for 10 min. The plasma was aliquoted and stored 12 to 15 months at −80°C until analysis of cytokines, gut-derived phenolics, and oxylipins. Blood samples were also collected into serum separator tubes, allowed to clot for 20 min, and then centrifuged for 10 min. Serum glucose (same day analysis) was analyzed by a commercial laboratory (Lab Corp, Burlington, NC). Enzyme-linked immunosorbent assays were used to measure plasma interleukin-6 (IL-6) and interleukin-1 receptor antagonist (IL-1ra) in duplicate in accordance with the manufacturer's protocols (R&D Systems, Inc., Minneapolis, Minnesota, USA). Within-subject samples were analyzed on the same assay plate to decrease inter-kit assay variability, and the intra-assay CV for both cytokines was <10%.

### Plasma Oxylipins

Plasma arachidonic acid (ARA), eicosapentaenoic acid (EPA), docosahexaenoic acid (DHA), and oxylipins were analyzed using a liquid chromatography-multiple reaction monitoring-mass spectrometry (LC-MRM-MS) method which measures 131 endogenous oxylipins, as fully described elsewhere (22). Resultant data files were processed with TraceFinder® 4.1 (ThermoFisher Scientific), and the auto-integrated peaks were inspected manually. Concentrations of each oxylipin were determined from calibration curves of each analyte, which was constructed by normalizing to the selected deuterated internal standards followed by linear regression with 1/x weighting. The coefficient of variation for the quality control standards was <15% as reported in the method development paper (22).

### Plasma Gut-Derived Phenolics

The broad-spectrum quantitative assay was optimized and validated to detect 229 analytes, which were quantified relative to 146 authentic commercial and synthetic standards (Data Sheet 1). Briefly, as previously described (23), metabolites were purified from 100 μL plasma by 96-well microelution 2-mg solid-phase extraction plates (SPE; Strata™-X, Phenomenex, Torrance, CA, USA). Extracts were separated and quantified via liquid chromatography tandem MS/MS, (ESI)−6500+ QTRAP (SCIEX, Framingham, MA, USA). Samples were injected onto a Kinetex PFP UPLC column (1.7 μm, 100Å, 100 mm, 2.1 mm ID; Phenomenex, Torrance, CA, USA). MS/MS scanning was achieved via advanced scheduled multiple-reaction monitoring (ADsMRM) using positive and negative ionization mode toggling in Analyst (Version 1.6.3, SCIEX, Framingham, MA, USA) with quantitation conducted using MultiQuant (Version 3.0.2, SCIEX, Framingham, MA, USA). Metabolites were confirmed on the basis of established retention times (using authentic and synthesized standards where possible) and three or more precursor-to-product ion transitions.

### Statistical Analysis

Except where otherwise indicated, data are expressed as mean ± standard error (*SE*). The study participant number (*n* = 15 per group) provided 80% power to detect a group difference with an effect size 1.06 at alpha 0.05 using two-sample *t*-tests. Gut phenolics and oxylipins variables were analyzed using mixed linear models with repeated measures, with gut-derived phenolics, or oxylipins as the response variable. Treatment (BLUE-WAT, BLUE-BAN, PLAC-WAT, PLAC-BAN), time (eight time points), and time × treatment (8 × 4) interactions were the independent variables. A random subject effect was also included in the model. Post-test contrasts for comparisons between different time points within each treatment, and between different treatments at each time point were performed. The positive false discovery rate (FDR or “*q*-value”) was calculated for multiple testing correction. Data were normalized as described in the methods. Missing values were imputed with half of the observed minimum after normalization. Data was log transformed to improve normality of the data. Outliers with Studentized residual >3 or < -3 were removed from the analysis. Q-Q plots were used to examine the distribution of the residues. Statistical significance was set at FDR adjusted *p* < 0.1 due to the large number of oxylipins and gut phenolics measured. Spearman correlation coefficients and the corresponding *P*-values were calculated between the serum glucose and the combined gut phenolics variables with the combined oxylipin variables at each time point across all of the study participants.

Orthogonal Partial Least Square Discriminant Analysis (OPLS-DA) in SIMCA (Version 14.1, Umetrics, Umeå, Sweden) was used to examine the overall pattern in the data. Metabolite perturbation was determined by measuring the distance of the loop (time 1 -> time 2 -> time 3 -> …-> time 8) in the OPLS-DA plot for each participant within each treatment. *T*-tests were used to compare the distance measured in each treatment group with the distance in PLAC-WAT group.

## Results

Characteristics of study participants and metabolic data collected during the 75-km cycling time trial are summarized in Table 1 for each group. No group differences were found. Three-days food records collected before the lab session with the 75-km cycling time trial revealed no significant differences in energy, carbohydrate, and micronutrient intake between groups (data not shown). For the entire group, energy intake averaged 2,292 ± 78 kcal/day (9.59 ± 0.33 MJ/day), with carbohydrate representing 45.1 ± 1.2% of total energy. Total flavonoid intake averaged 96.9 ± 17.1 mg/day (excluding the blueberry supplement).

**Table 1:** Characteristics of study participants that completed all aspects of the study (*n* = 59) and 75-km cycling physiological parameters^*^.

**Variable mean ±*SE***	**Placebo—Water**	**Blueberry—Water**	**Placebo—Banana**	**Blueberry—Banana**
	**(*n* = 15)**	**(*n* = 15)**	**(*n* = 14)**	**(*n* = 15)**
	**(11 males, 4 females)**	**(10 males, 5 females)**	**(9 males, 5 females)**	**(10 males, 5 females)**
Age (years)	39 ± 2	38 ± 2	39 ± 2	38 ± 2
Height (cm)	177 ± 2	176 ± 2	175 ± 3	174 ± 2
Weight (kg)	76.6 ± 3.5	74.6 ± 3.8	74.8 ± 3.9	70.4 ± 2.9
Body fat (%)	21.3 ± 2.1	19.6 ± 2.3	21.1 ± 1.5	18.0 ± 1.5
Maximal watts	292 ± 11.1	307 ± 11.6	288 ± 12.2	298 ± 12.6
VO_2max_ (ml^.^kg^−1.^min^−1^)	47.6 ± 1.8	49.9 ± 2.4	45.7 ± 1.6	50.0 ± 1.8
Maximal heart rate (beats/min)	175 ± 2.5	172 ± 2.6	178 ± 2.8	180 ± 3.0
**75-km Cycling Bout**				
Watts	147 ± 10	161 ± 9	152 ± 10	154 ± 11
VO_2_ (L/min)	2.41 ± 0.11	2.50 ± 0.12	2.46 ± 0.09	2.39 ± 0.09
Heart rate	144 ± 3	144 ± 3	149 ± 3	151 ± 3
Total time (minutes)	195 ± 8.5	178 ± 6.6	186 ± 6.2	183 ± 7.9

* *Groups did not differ significantly on any of the listed variables*.

The average participant took 3.09 ± 12 h to complete the difficult 75-km cycling course with high relative heart rates (83.4 ± 1.5% maximal heart rate) and oxygen consumption (69.5 ± 2.0% maximal oxygen consumption rate or VO_2max_). Serum glucose was elevated immediately post-exercise in the participants consuming bananas (PLAC-BAN and BLU-BAN) (111 ± 3.2 mg/dL) compared to those drinking water (BLU-WAT and PLAC-WAT) (82.6 ± 3.8 mg/dL) (*p* < 0.001). Post-exercise plasma levels of IL-6 and IL-1ra were lower in participants consuming bananas (12.5 ± 1.8, 1,330 ± 368 pg/mL) compared to those drinking water (16.1 ± 1.5, 2,823 ± 432 pg/mL), but the trial difference was significant only for IL-1ra (*p* = 0.141, 0.012, respectively).

**Figure 2:**
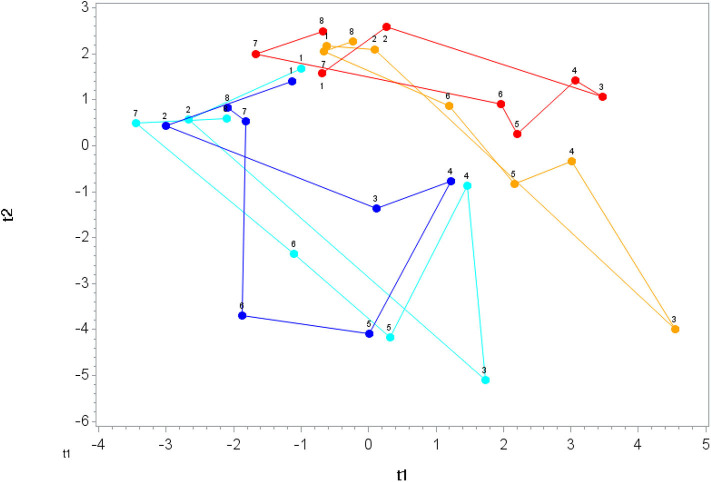
Orthogonal partial least squares discriminant analysis (OPLS-DA) graph for the gut phenolics data. The centroid of each treatment at each time point (1, 2, 3, …, 8) was calculated, and loops were formed and lengths measured for each treatment. The loop length for PLAC-WAT (red line) (control) was the shortest (16.3 ± 2.3) and was significantly shorter than BLUE-BAN (light blue line) (29.2 ± 2.1) (*p* = 0.0001) and BLUE-WAT (dark blue line) (22.4 ± 2.0) (*p* = 0.049), but not different from PLAC-BAN (gold line) (20.5 ± 2.1) (*p* = 0.182).

**Figure 3:**
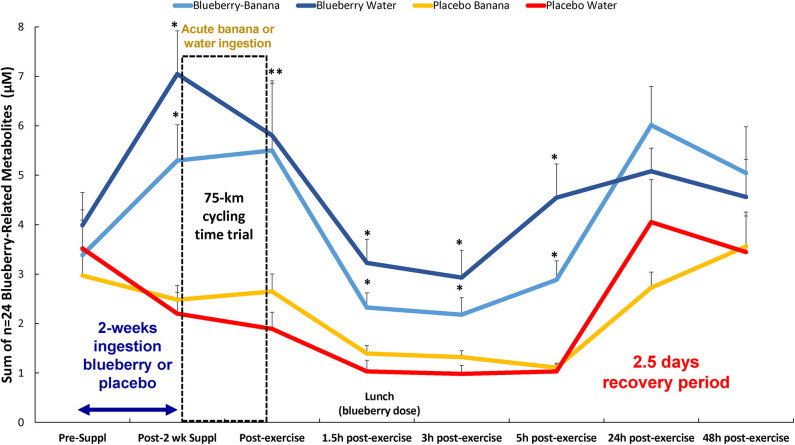
Change in the sum of *n* = 24 plasma blueberry-related metabolites in each of the four groups over time. Time × treatment effect, *p* < 0.001; treatment effect, *p* < 0.001. * FDR adjusted *p* < 0.05 relative to PLAC-WAT. The composition of the lunch is described in the methods section. Data are expressed as mean ± *SE*. **Significance for both blueberry groups.

A total of 166 gut-derived phenolics were identified from the plasma samples. OPLS-DA showed distinct group differences in metabolite perturbation between the PLAC-WAT and BLUE-BAN (*p* < 0.001) and BLUE-WAT (*p* = 0.049) groups, but not with the PLAC-BAN group (*p* = 0.182) (Figure 2). Of the 166 gut-derived phenolics, significant time × treatment effects were found for 45 (Data Sheet 1), with 24 of these associated with blueberry intake (when contrasting the BLUE-WAT and PLAC-WAT groups) (Table 2). These 24 metabolites were grouped and graphed, with significant elevations shown for the BLUE-WAT and BLUE-BAN groups compared to PLAC-WAT after 2-weeks supplementation and for 5 h post-exercise (Figure 3) (time × treatment effect, FDR adjusted *p* < 0.001). Acute banana ingestion was associated with post-exercise elevations in seven metabolites including 2-isopropylalmalate, 4-hydroxy-3,5-dimethoxybenzoic acid, 3-hydroxy-4-methoxycinnamic acid sulfate, 5-hydroxyindoleacetate, 3-(3,4,5-trimethoxyphenyl)propanoic acid, 4-hydroxy-3,5-dimethoxycinnamic acid, and dopamine 3-sulfate. As a group, these seven banana-related metabolites increased from 0.052 ± 0.005 to 0.85 ± 0.10 μM in the two banana groups combined compared to no change in the other two groups drinking water alone during exercise, with these group differences largely erased by 5 h post-exercise (time × treatment effect, FDR adjusted *p* < 0.001) (Data Sheet 1).

**Table 2:** Blueberry-related metabolites with significant interaction effects [time (eight time points) × treatment (four groups)].

**Blueberry-related gut phenolic**	**Interaction effect, FDR**
	**adjusted *p* < 0.05**
4-hydroxy-3,5-dimethoxybenzoic acid	< 0.001
3-hydroxy-4-methoxycinnamic acid sulfate	< 0.001
Hydroxybenzoic acid-sulfate (isomer 1)	< 0.001
Hippuric acid	< 0.001
3-methoxybenzoic acid-4-O-glucuronide	< 0.001
3-hydroxyhippuric acid	< 0.001
3-methoxybenzoic acid-4-O-glucuronide	< 0.001
4-hydroxy-3,5-dimethoxycinnamic acid	< 0.001
3-methoxybenzoic acid 4-sulfate	< 0.001
Methoxycinnamic acid-O-glucuronide	< 0.001
4-hydroxyphenylacetic acid	< 0.001
3-hydroxyphenylacetic acid	< 0.001
3,4-dihydroxycinnamic acid sulfate (isomer 1)	0.001
3-(4-hydroxyphenyl)propanoic acid	0.003
3-hydroxy-4-methoxyphenylacetic acid	0.004
Hydroxyhippuric acid (isomer 1)	0.007
3-(3-hydroxy-4-methoxyphenyl)propanoic acid	0.007
Hydroxy-methoxyphenylacetic acid-O-glucuronide	0.007
Hydroxyhippuric acid (isomer 3)	0.009
Methoxycinnamic acid-sulfate	0.009
4-hydroxy-3,5-dimethoxyphenylacetic acid	0.019
3-(4-hydroxy-3-methoxyphenyl)propanoic acid	0.023
3-O-caffeoylquinic acid	0.025
Hydroxybenzoic acid-sulfate (isomer 3)	0.032

**Figure 4:**
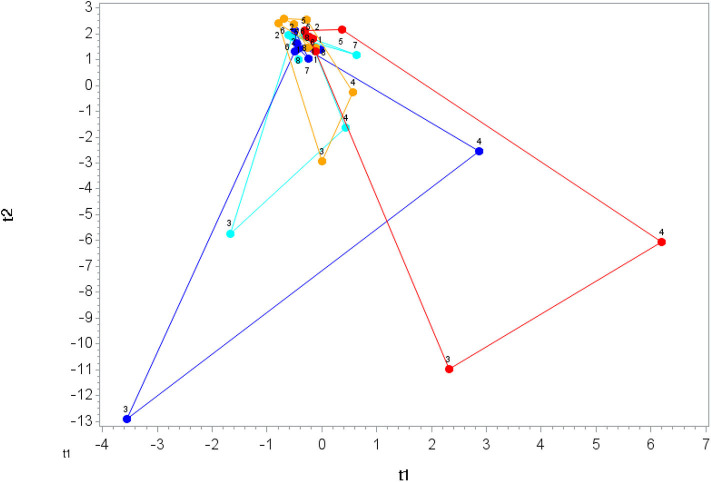
Orthogonal partial least squares discriminant analysis (OPLS-DA) graph for the oxylipin data. The centroid of each treatment at each time point (1, 2, 3, …, 8) was calculated, and loops were formed and lengths measured for each treatment. The loop length for PLAC-WAT (red line) (control) was the longest (38.1 ± 5.8) and was significantly longer than PLAC-BAN (gold line) (17.3 ± 5.8) (*p* = 0.015) but not different from BLUE-BAN (light blue line) (28.1 ± 5.5) (*p* = 0.221) and BLUE-WAT (dark blue line) (37.9 ± 6.1) (*p* = 0.982).

The 75-km cycling bout increased plasma levels for ARA, DHA, and EPA (Data Sheet 2) and 64 of 67 identified oxylipins (Table 3). Mean fold increases for the 64 oxylipins were 11.8 ± 13.9 (mean ± *SD*) immediately post-exercise, 6.5 ± 5.3 1.5 h post-exercise, and 1.04 ± 1.14 3 h post-exercise (Data Sheet 2). OPLS-DA showed group differences in oxylipin perturbation between the PLAC-WAT and PLAC-BAN groups (*p* = 0.015), but not with the BLUE-WAT (*p* = 0.982) and BLUE-BAN (*p* = 0.221) groups (Figure 4). Of the 67 identified oxylipins, 15 (22%) had time × treatment effects with FDR adjusted *p* ≤ 0.05, and 20 (30%) with FDR adjusted *p* ≤ 0.10 (Data Sheet 2).

**Table 3:** Time effects for plasma oxylipins, with 64 of 67 increasing post-exercise. Oxylipins are categorized by substrate and enzyme systems.

**Substrate**	**Enzyme**	**Name**	**Time effect, FDR**
			**adjusted *p*-value**
AA	COX	dihomo PGE2	< 0.001
ALA	LOX	9-HOTrE	< 0.001
ALA	LOX	13-HOTrE	< 0.001
ARA	COX	12-HHTrE	< 0.001
ARA	COX	PGE2	< 0.001
ARA	COX	TxB2	< 0.001
ARA	COX	bicyclo PGE2	0.833
ARA	COX/Non-enzymatic	PGFM	< 0.001
ARA	CYP	11,12-EET	< 0.001
ARA	CYP	11,12-diHETrE	< 0.001
ARA	CYP	18-HETE	< 0.001
ARA	CYP	20-HETE	< 0.001
ARA	CYP	19-HETE	< 0.001
ARA	CYP	14,15-diHETrE	< 0.001
ARA	CYP	5,15-diHETE	< 0.001
ARA	CYP	8,9-diHETrE	< 0.001
ARA	CYP	5,6-EET	< 0.001
ARA	CYP	16-HETE	< 0.001
ARA	CYP	17-HETE	< 0.001
ARA	CYP	5,6-diHETrE	0.006
ARA	LOX	15-HETE	< 0.001
ARA	LOX	12-HETE	< 0.001
ARA	LOX	tetranor 12-HETE	< 0.001
ARA	LOX	5-HETE	< 0.001
ARA	LOX	11-HETE	< 0.001
ARA	LOX	8-HETE	< 0.001
ARA	LOX	9-HETE	< 0.001
ARA	LOX	15R-LXA4	0.002
ARA	LOX	5-oxoETE	0.007
ARA	LOX	12-oxoETE	0.008
ARA	LOX	6S-LXA4	0.428
ARA	Non-enzymatic	8,12-iso-Isoprostane-F2α-VI	< 0.001
ARA	Non-enzymatic	13,14-Dihydro-15-keto PGF2a	< 0.001
DGLA	COX	TxB1	0.002
DGLA	LOX	5-HETrE	< 0.001
DGLA	LOX	15-HETrE	< 0.001
DGLA	LOX	8-HETrE	< 0.001
DHA	CYP	20-HDoHE	< 0.001
DHA	CYP	19,20-DiHDPA	< 0.001
DHA	CYP	20cooh AA	< 0.001
DHA	LOX	4-HDoHE	< 0.001
DHA	LOX	10-HDoHE	< 0.001
DHA	LOX	16-HDoHE	< 0.001
DHA	LOX	14-HDoHE	< 0.001
DHA	LOX	11-HDoHE	< 0.001
DHA	LOX	13-HDoHE	< 0.001
DHA	LOX	Protectin D1	< 0.001
DHA	LOX	17 HDoHE	< 0.001
DHA	LOX	7-HDoHE	< 0.001
DHA	LOX	8-HDoHE	0.003
EPA	COX	TxB3	< 0.001
EPA	COX	PGE3	< 0.001
EPA	COX	18-HEPE	0.015
EPA	LOX	12-HEPE	< 0.001
EPA	LOX	9-HEPE	< 0.001
EPA	LOX	8-HEPE	< 0.001
EPA	LOX	5-HEPE	< 0.001
EPA	LOX	15-HEPE	< 0.001
EPA	LOX	11-HEPE	< 0.001
EPA	LOX	Resolvin E1	0.024
LA	CYP	12,13-DiHOME	< 0.001
LA	CYP	9,10-DiHOME	< 0.001
LA	CYP	9,10-EpOME	0.851
LA	LOX	9-HODE	< 0.001
LA	LOX	13-HODE	< 0.001
LA	LOX	9-oxoODE	< 0.001
LA	LOX	13-oxoODE	< 0.001

*AA, adrenic acid; ALA, alpha-linolenic acid; ARA, arachidonic acid; DGLA, dihomo-gamma-linolenic acid; DHA, docosahexaenoic acid; EPA, Eicosapentaenoic acid; LA, linoleic acid; COX, cyclooxygenase; CYP, cytochrome P450; LOX, lipoxygenase; 20cooh AA, 20-carboxy-arachidonic acid; DiHDPA, dihydroxy-docosapentaenoic acid; DiHETE, dihydroxy-eicosatetraenoic acid; DiHETrE, dihydroxy-eicosatrienoic acid; DiHOME, dihydroxy-9Z-octadecenoic acid; EET, epoxyeicosatrienoic acid; EpOME, epoxyoctadecenoic acid; HDoHE, hydroxy-docosahexaenoic acid; HEPE, hydroxy-eicosapentaenoic acid; HETE, hydroxy-eicosatetraenoic acid; HHTrE, hydroxy-heptadecatrienoic acid; HODE, hydroxy-octadecadienoic acid; HOTrE, hydroxy-octadecatrienoic acid; Lx, lipoxin; oxoETE, oxo-eicosatetraenoic acid; oxo-ODE, oxo-octadecadienoic acid; PG, prostaglandin; TxB, thromboxane*.

**Figure 5:**
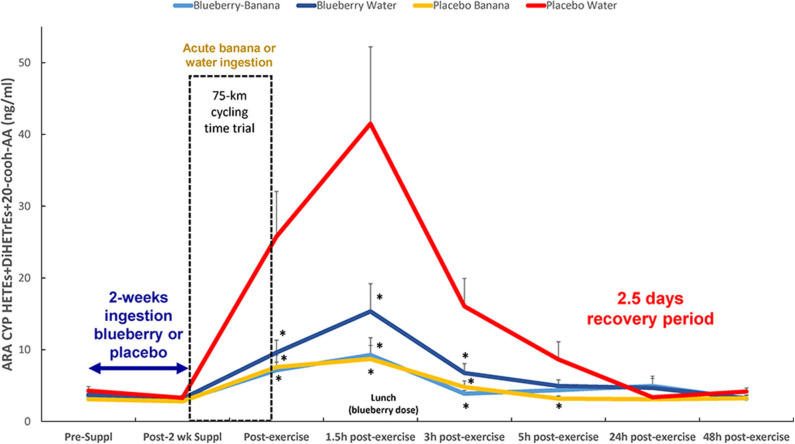
Post-exercise levels of plasma ARA-CYP HETEs, 20-cooh-AA (stable 20-HETE metabolite), and diHETrEs were attenuated with acute carbohydrate and 2-weeks blueberry intake. Time × treatment effect, *p* = 0.127; treatment effect, *p* = 0.003; *FDR adjusted *p* < 0.10 relative to PLAC-WAT. The composition of the lunch is described in the methods section. Data are expressed as mean ± *SE*.

Based on these results, the oxylipins were grouped according to relevant substrates and enzyme systems (Table 4), with *post-hoc* probing for group differences when FDR adjusted *p* < 0.05 for either the treatment or time × treatment effect. The most meaningful results from this analysis were with the group of 10 ARA-CYP pro-inflammatory oxylipins: HETEs (16-,17-,18-,19-,20-HETE, 20cooh AA) and diHETrEs (5,6-, 8,9-, 11,12-, and 14,15-diHETrEs) (treatment effect, *p* = 0.003) (Figure 5). This analysis showed that acute carbohydrate ingestion from bananas during exercise was associated with a 4.6-fold decrease in plasma levels of these ARA CYP oxylipins at 1.5 h post-exercise, with blueberry ingestion associated with 2.7-fold decrease. These group differences were no longer apparent by 5 h post-exercise. Acute carbohydrate ingestion decreased plasma levels of three fatty acid substrates (sum of ARA, DHA, and EPA) immediately post-exercise by 35% (FDR adjusted *p* < 0.05) when comparing the two banana groups with the two water-only groups (Data Sheet 2).

**Table 4:** Oxylipin groups: treatment, and time × treatment interaction effects [time (eight time points) × treatment (four groups)]^*^.

**Oxylipin group**	**Treatment**,	**Time x treatment**,
	**FDR adjusted**	**FDR adjusted**
	***p*-value**	***p*-value**
ALA-LOX (9+13-HOTrE)	0.0238	0.0661
ARA-CYP DiHETrEs (5,6-, 8,9-, 11,12-, 14,15-)	0.0289	0.0170
ARA-CYP HETES (16-,17-,18-,19-, 20-HETE)	0.0465	0.4361
ARA-CYP HETEs (16–20), DiHETrEs, 20-coohAA	0.0031	0.1275
ARA-LOX HETEs (5-,8-,9-,11-,12-,15-HETE)	0.1281	0.4873
DHA-CYP (20-HDoHE, 19,20-DiHDPA)	0.0238	0.0181
DHA-LOX HDoHEs (4-,7-,8-,10-,11-,13-,14-,16-,17-HDoHE)	0.0238	0.7332
EPA-LOX HEPEs (5-,8-,9-,11-,12-,15-HEPE)	0.0238	0.6019
LA-LOX (9+13-HODE)	0.0465	0.1251
LA-CYP (9,10- + 12,13-DiHOME)	0.1499	0.5080

* *See footnote at Table 3 for abbreviations*.

The Spearman correlation analysis showed a negative relationship between immediate-post-exercise levels of serum glucose and plasma levels of the group of 10 ARA-CYP pro-inflammatory oxylipins (*rs* = −0.43, *p* = 0.001). A negative relationship between 1.5 h post-exercise plasma levels of blueberry metabolites (group of 24) and plasma levels of the 10 ARA-CYP oxylipins was also shown (*rs* = −0.29, *p* = 0.038). Immediate- and 1.5 h-post-exercise plasma IL-6 levels were significantly correlated with the group of 10 ARA-CYP pro-inflammatory oxylipins (*rs* = 0.038, *p* = 0.005, and *rs* = 0.42, *p* = 0.001, respectively).

**Figure 6:**
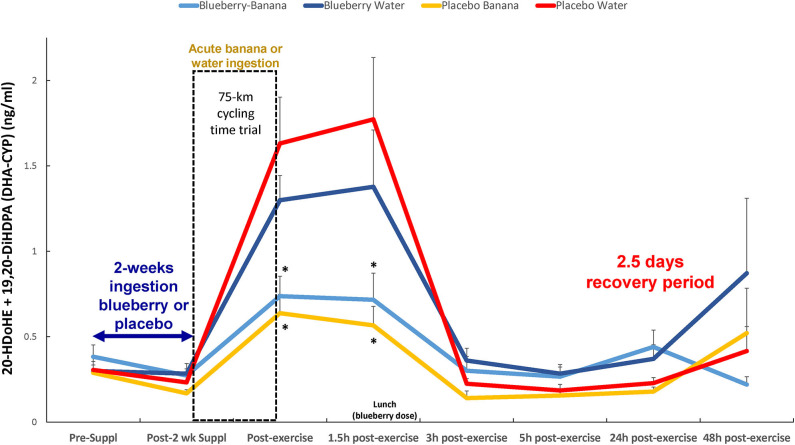
Post-exercise levels of plasma DHA-CYP oxylipins (20-HDoHE, 19,20-DiHDPA) were lower with acute carbohydrate ingestion from bananas, but not 2-weeks blueberry intake, compared to the PLAC-WAT group (time × treatment effect, FDR adjusted *p* = 0.018; treatment effect, FDR adjusted *p* = 0.024). *FDR adjusted *p* < 0.10 relative to PLAC-WAT. The composition of the lunch is described in the methods section. Data are expressed as mean ± *SE*.

Another combined analysis of two DHA-CYP oxylipins (20-HDoHE, 19,20-DiHDPA) showed that acute carbohydrate ingestion from bananas, but not 2-weeks ingestion of blueberries, was associated with lower immediate- and 1.5 h-post-exercise plasma levels (2.76-fold at 1.5 h recovery) (time × treatment effect, *p* = 0.018; treatment effect, *p* = 0.024) (Figure 6). The Spearman correlation analysis showed a negative relationship between immediate-post-exercise levels of serum glucose and plasma levels of these DHA-CYP oxylipins (*rs* = −0.45, *p* < 0.001).

## Discussion

This study confirmed that a large number of diverse oxylipins are generated in response to prolonged and intensive exercise in cyclists (5). Plasma levels of 10 pro-inflammatory oxylipins derived from the ARA-CYP pathway were strongly attenuated with acute banana ingestion, similar to what we showed previously (5). For the first time we observed that 2-weeks blueberry ingestion at a moderate level (one cup equivalent per day, 1,059 mg total polyphenols) increased plasma levels of 24 gut-derived phenolics and was associated with lowered post-exercise levels of ARA-CYP oxylipins. This interpretation was strengthened by the Spearman correlation analysis that showed negative relationships post-exercise for serum glucose levels or plasma blueberry metabolites with the group of 10 ARA-CYP oxylipins.

Oxylipins are synthesized from cell membrane phospholipid PUFAs as they are released under tight regulation by phospholipase A2 (PLA2) in response to cell activation from various stress-related stimuli including exercise (12, 24, 25). COX, LOX, and CYP enzyme systems metabolize the released PUFAs into oxylipins that act as autocrine and paracrine lipid mediators in numerous physiological processes (12, 13). Depending on the metabolic context, oxylipins can function as beneficial signaling agents or mediators of inflammation, immune dysfunction, and disease (13, 26).

The oxylipin response to exercise is a new area of scientific endeavor, and little is known regarding their physiological roles during recovery or the influence of dietary interventions (5, 27–30). Nearly half of the plasma oxylipins elevated in response to 75-km cycling came from the substrate ARA, and many of these were generated from the CYP enzyme system. These ARA-CYP oxylipins included the terminal/subterminal HETEs: 16-, 17-, 18-, 19-, and 20-HETE, and the stable 20-HETE metabolite 20-cooh-AA. The terminal/subterminal HETEs are generated from specific types of CYPs (CYP1As, CYP4As, CYP4Fs), and are regarded as pro-inflammatory (31–34). Terminal/subterminal HETEs are involved in various diseases including hypertension, acute coronary syndrome, diabetic retinopathy, non-alcoholic fatty liver disease, ischemic stroke, and inflammatory diseases, and elucidation of underlying mechanisms may lead to new therapeutic approaches (34).

Other types of CYPs (CYP2Bs, CYP2Cs, CYP2Js) oxidize ARA into epoxyeicosatrienoic acids (EETs) that can be further oxidized to DiHETrEs when soluble epoxide hydrolase (sEH) is activated (35–38). EETs are disease-protective, anti-inflammatory oxylipins in contrast to the DiHETrEs that exert pro-inflammatory effects (35). Our data indicate the 10 ARA-CYP, pro-inflammatory oxylipins and 5,6-EET increased to high levels for several hours post-exercise in overnight fasted cyclists from the PLAC-WAT group. Acute carbohydrate ingestion from bananas or 2-weeks blueberry ingestion countered post-exercise increases in 5,6-EET and this group of ARA-CYP oxylipins. These data suggest that PLA2 and CYP enzymes, but perhaps not sEH, may be influenced by shifts in blood levels of glucose, blueberry metabolites, and related co-factors during prolonged exercise stress. Mobilization of ARA, DHA, and EPA immediately post-exercise was decreased 35% with carbohydrate intake from bananas, suggesting that PLA2 enzymes may be downregulated when blood glucose is elevated. The decrease in ARA, DHA, and EPA substrates with carbohydrate intake during exercise may have influenced oxylipin generation from these fatty acids. However, published data in support of these findings are lacking.

At least 57 human genes code for CYP enzymes, and expression is regulated by multiple factors including miRNAs, cytokines, hormones, and xenobiotics (35–38). We are unaware of published data supporting a negative relationship between blood glucose and ARA-CYP oxylipin generation in response to carbohydrate feeding during intensive exercise. Up regulation of 20-HETE CYP isoforms has been reported with glucose deprivation in cell cultures (39). Our previous study showed no difference in the post-exercise mitigation of ARA-CYP oxylipins when consuming bananas or a 6% sugar beverage (5). These data suggest that specific banana metabolites that increased strongly but in low concentrations during prolonged exercise may have little influence on CYP enzymes and sEH. Carbohydrate ingestion during prolonged and intensive exercise attenuates plasma levels of IL-6 and IL-1ra, and this was confirmed for IL-1ra but not IL-6 in this study (1–4). Limited data indicate that CYP activity is increased when IL-6 is elevated as supported by our positive relationship between the ARA-CYP oxylipins and plasma IL-6 during early recovery (40, 41).

Cell culture data suggest that certain types of CYP enzymes and sEH are inhibited through flavonoid-ligand effects, but these findings have limited utility because the parent flavonoid molecules are extensively biotransformed after ingestion (16–20). Study participants in BLUE-WAT and BLUE-BAN did not consume their daily blueberry dose until 1.5 h post-exercise (at lunch) to ensure that no carbohydrate was consumed prior to the cycling bout. Thus, the post-exercise ARA-CYP lowering effect was related to elevated plasma levels of blueberry gut-derived phenolics. This is a novel finding that will require confirmation in cell culture studies using the *in vivo* blueberry gut-derived phenolics identified in this study. The ARA-CYP lowering effect of blueberry ingestion was modest in comparison to acute carbohydrate intake from bananas, and the combination of blueberry and banana intake was not additive.

Plasma levels of DHA-CYP oxylipins (20-HDoHE and 19,20-DiHDPA) increased strongly post-exercise in PLAC-WAT and BLUE-WAT, with significantly lower levels in those ingesting carbohydrate from bananas. Limited evidence suggests that 20-HDoHE exerts anti-inflammatory effects and may be generated when oxidative stress and pro-inflammatory conditions are present (42). The dihydroxy oxylipin 19,20-DiHDPA is also regarded as anti-inflammatory, and is elevated with long-term niacin treatment (43). As previously reported and confirmed in this study, carbohydrate ingestion reduces plasma levels of both pro- and anti-inflammatory cytokines, and is analogous to the reduction in both pro-inflammatory ARA-CYP and anti-inflammatory DHA-CYP oxylipins (1–6, 15).

## Conclusions

This study verified that exercise-induced physiological stress is associated with large-fold increases in plasma oxylipins in overnight fasted cyclists who consume only water (5). Acute carbohydrate ingestion from bananas had a strong countermeasure effect, especially in lowering post-exercise levels of oxylipins generated from ARA- and DHA-CYP. Blueberry ingestion (1 cup per day) for just 2 weeks increased overnight-fasted blood levels of numerous gut-derived phenolic metabolites in the cyclists. There is a growing awareness that these phenolic metabolites confer wide-ranging physiological effects, and we reasoned that blueberry ingestion may influence oxylipin generation during exercise (8, 44, 45). In agreement with our hypothesis, cyclists exercising vigorously for about 3 h with these blueberry phenolic metabolites in their systems had lower plasma levels of ARA-CYP oxylipins during several hours of recovery. ARA-CYP oxylipins have fundamental roles in physiology and pathophysiology, especially in mediating inflammatory and innate immune responses to physiological stress (12, 13, 25, 26). Although much progress has been made during the past decade regarding oxylipin metabolism, few data are available to elucidate the influence of glucose and blueberry metabolites on PLA2, CYP, and sEH activity within an exercise stress context.

Concerns have been expressed that carbohydrate and antioxidant intake before, during, and after intensive and prolonged exercise may interfere with signaling pathways for training adaptations, but the available data do not provide a clear consensus (46–48). Our data indicate that banana and/or blueberry ingestion do not completely counter inflammatory-related oxylipins, but rather attenuate the magnitude. Future research will help determine if the post-exercise reduction in 5,6-EET and pro-inflammatory ARA-CYP oxylipins with banana or blueberry ingestion confers long-term performance and health benefits for the athlete.

## Data Availability Statement

The raw data supporting the conclusions of this article will be made available by the authors, without undue reservation.

## Ethics Statement

Participants signed the informed consent form and study procedures were approved by the Institutional Review Board at Appalachian State University.

## Author Contributions

DN, NG, CK, and ML designed research. DN, NG, and ML conducted research. DN, NG G-YC, QZ, WS, CK, PC, KK, and ML analyzed samples and conducted data analysis. DN, NG, CK, and ML wrote the paper. DN had primary responsibility for final content. All authors read and approved the final manuscript.

## Conflict of Interest

The authors declare that the research was conducted in the absence of any commercial or financial relationships that could be construed as a potential conflict of interest.

## Supplementary Material

Checklist S1 CONSORT checklist.

Data Sheet 1 Identified gut-derived phenolics and standards library.

Data Sheet 2 Identified oxylipins.

## References

[1] Nieman DC Gillitt ND Henson DA Sha W Shanely A Knab AM. Bananas as an energy source during exercise: a metabolomics approach. PLoS ONE. (2012) 7:e37479. 10.1371/journal.pone.0037479 22616015 PMC3355124

[2] Nieman DC Gillitt ND Sha W Meaney MP John C Pappan KL. Metabolomics-based analysis of banana and pear ingestion on exercise performance and recovery. J Proteome Res. (2015) 14:5367–77. 10.1021/acs.jproteome.5b00909 26561314

[3] Nieman DC Davis JM Henson DA Walberg-Rankin J Shute M Dumke CL. Carbohydrate ingestion influences skeletal muscle cytokine mRNA and plasma cytokine levels after a 3-h run. J Appl Physiol. (2003) 94:1917–25. 10.1152/japplphysiol.01130.2002 12533503

[4] Nieman DC Gillitt ND Sha W Esposito D Ramamoorthy S Metabolic recovery from heavy exertion following banana compared to sugar beverage or water only ingestion: a randomized, crossover trial. PLoS ONE. (2018) 13:e0194843 10.1371/journal.pone.0194843 29566095 PMC5864065

[5] Nieman DC Gillitt ND Chen GY Zhang Q Sakaguchi CA Stephan EH. Carbohydrate intake attenuates post-exercise plasma levels of cytochrome P450-generated oxylipins. PLoS ONE. (2019) 14:e0213676. 10.1371/journal.pone.0213676 30883596 PMC6422332

[6] Grace MH Xiong J Esposito D Ehlenfeldt M Lila MA. Simultaneous LC-MS quantification of anthocyanins and non-anthocyanin phenolics from blueberries with widely divergent profiles and biological activities. Food Chem. (2019) 277:336–46. 10.1016/j.foodchem.2018.10.101 30502155 PMC6287264

[7] Nieman DC Gillitt ND Knab AM Shanely RA Pappan KL Jin F. Influence of a polyphenol-enriched protein powder on exercise-induced inflammation and oxidative stress in athletes: a randomized trial using a metabolomics approach. PLoS ONE. (2013) 8:e72215. 10.1371/journal.pone.0072215 23967286 PMC3744465

[8] Curtis PJ van der Velpen V Berends L Jennings A Feelisch M Umpleby AM. Blueberries improve biomarkers of cardiometabolic function in participants with metabolic syndrome-results from a 6-month, double-blind, randomized controlled trial. Am J Clin Nutr. (2019) 109:1535–45. 10.1093/ajcn/nqy380 31136659 PMC6537945

[9] de Ferrars RM Czank C Zhangm Q Botting NP Kroon PA Cassidy A. The pharmacokinetics of anthocyanins and their metabolites in humans. Br J Pharmacol. (2014) 171:3268–82. 10.1111/bph.12676 24602005 PMC4080980

[10] Kay CD Pereira-Caro G Ludwig IA Clifford MN Crozier A. Anthocyanins and flavanones are more bioavailable than previously perceived: a review of recent evidence. Annu Rev Food Sci Technol. (2017) 8:155–80. 10.1146/annurev-food-030216-025636 28125348

[11] Chandra P Rathore AS Kay KL Everhart JL Curtis P Burton-Freeman B. Contribution of berry polyphenols to the human metabolome. Molecules. (2019) 24:4220. 10.3390/molecules24234220 31757061 PMC6930569

[12] Gabbs M Leng S Devassy JG Monirujjaman M Aukema HM. Advances in our understanding of oxylipins derived from dietary PUFAs. Adv Nutr. (2015) 6:513–40. 10.3945/an.114.007732 26374175 PMC4561827

[13] Caligiuri SPB Parikh M Stamenkovic A Pierce GN Aukema HM. Dietary modulation of oxylipins in cardiovascular disease and aging. Am J Physiol Heart Circ Physiol. (2017) 313:H903–8. 10.1152/ajpheart.00201.2017 28801523

[14] Ostermann AI Schebb NH. Effects of omega-3 fatty acid supplementation on the pattern of oxylipins: a short review about the modulation of hydroxy-, dihydroxy-, and epoxy-fatty acids. Food Funct. (2017) 8:2355–67. 10.1039/C7FO00403F 28682409

[15] Nieman DC Lila MA Gillitt ND. Immunometabolism: a multi-omics approach to interpreting the influence of exercise and diet on the immune system. Annu Rev Food Sci Technol. (2019) 10:341–63. 10.1146/annurev-food-032818-121316 30633566

[16] Bai MM Shi W Tian JM Lei M Kim JH Sun YN. Soluble epoxide hydrolase inhibitory and anti-inflammatory components from the leaves of Eucommia ulmoides Oliver (duzhong). J Agric Food Chem. (2015) 63:2198–205. 10.1021/acs.jafc.5b00055 25679330

[17] Dutkiewicz Z Mikstacka R. Structure-based drug design for cytochrome P450 family 1 inhibitors. Bioinorg Chem Appl. (2018) 2018:3924608. 10.1155/2018/3924608 30147715 PMC6083639

[18] Hoek-van den Hil EF van Schothorst EM van der Stelt I Swarts HJ van Vliet M Amolo T. Direct comparison of metabolic health effects of the flavonoids quercetin, hesperetin, epicatechin, apigenin and anthocyanins in high-fat-diet-fed mice. Genes Nutr. (2015) 10:469. 10.1007/s12263-015-0469-z 26022682 PMC4447677

[19] Takemura H Itoh T Yamamoto K Sakakibara H Shimoi K. Selective inhibition of methoxyflavonoids on human CYP1B1 activity. Bioorg Med Chem. (2010) 18:6310–5. 10.1016/j.bmc.2010.07.020 20696580

[20] Liu J Sridhar J Foroozesh M. Cytochrome P450 family 1 inhibitors and structure-activity relationships. Molecules. (2013) 18:14470–95. 10.3390/molecules181214470 24287985 PMC4216474

[21] Singleton VL Orthofer R Lamuela-Raventos RM Analysis of total phenols and other oxidation substrates and antioxidants by means of folin-ciocalteu reagent. Methods Enzymol. (1999) 299:152–78. 10.1016/S0076-6879(99)99017-1

[22] Chen GY Zhang Q. Comprehensive analysis of oxylipins in human plasma using reversed-phase liquid chromatography-triple quadrupole mass spectrometry with heatmap-assisted selection of transitions. Anal Bioanal Chem. (2019) 411:367–85. 10.1007/s00216-018-1446-3 30406832 PMC6457987

[23] Nieman DC Kay CD Rathore AS Grace MH Strauch RC Stephan EH. Increased plasma levels of gut-derived phenolics linked to walking and running following two weeks of flavonoid supplementation. Nutrients. (2018) 10:1718. 10.3390/nu10111718 30423955 PMC6267437

[24] Guijas C Rodríguez JP Rubio JM Balboa MA Balsinde J. Phospholipase A2 regulation of lipid droplet formation. Biochim Biophys Acta. (2014) 1841:1661–71. 10.1016/j.bbalip.2014.10.004 25450448

[25] Astudillo AM Balboa MA Balsinde J. Selectivity of phospholipid hydrolysis by phospholipase A2 enzymes in activated cells leading to polyunsaturated fatty acid mobilization. Biochim Biophys Acta Mol Cell Biol Lipids. (2019) 1864:772–83. 10.1016/j.bbalip.2018.07.002 30010011

[26] Shearer GC Walker RE. An overview of the biologic effects of omega-6 oxylipins in humans. Prostaglandins Leukot Essent Fatty Acids. (2018) 137:26–38. 10.1016/j.plefa.2018.06.005 30293594

[27] García-Flores LA Medina S Gómez C Wheelock CE Cejuela R Martínez-Sanz JM. Aronia-citrus juice (polyphenol-rich juice) intake and elite triathlon training: a lipidomic approach using representative oxylipins in urine. Food Funct. (2018) 9:463–75. 10.1039/C7FO01409K 29231216

[28] Vella L Markworth JF Farnfield MM Maddipati KR Russell AP Cameron-Smith D. Intramuscular inflammatory and resolving lipid profile responses to an acute bout of resistance exercise in men. Physiol Rep. (2019) 7:e14108. 10.14814/phy2.14108 31257737 PMC6599756

[29] Markworth JF Vella L Lingard BS Tull DL Rupasinghe TW Sinclair AJ. Human inflammatory and resolving lipid mediator responses to resistance exercise and ibuprofen treatment. Am J Physiol Regul Integr Comp Physiol. (2013) 305:R1281–96. 10.1152/ajpregu.00128.2013 24089379 PMC3882565

[30] Markworth JF D'Souza RF Aasen KMM Mitchell SM Durainayagam BR Sinclair AJ. Arachidonic acid supplementation transiently augments the acute inflammatory response to resistance exercise in trained men. J Appl Physiol (1985). (2018). 125:271–86. 10.1152/japplphysiol.00169.2018 29698111

[31] Rocic P Schwartzman ML. 20-HETE in the regulation of vascular and cardiac function. Pharmacol Ther. (2018) 192:74–87. 10.1016/j.pharmthera.2018.07.004 30048707 PMC6278600

[32] Waldman M Peterson SJ Arad M Hochhauser E. The role of 20-HETE in cardiovascular diseases and its risk factors. Prostaglandins Other Lipid Mediat. (2016) 125:108–17. 10.1016/j.prostaglandins.2016.05.007 27287720

[33] Hoxha M Zappacosta B. CYP-derived eicosanoids: implications for rheumatoid arthritis. Prostaglandins Other Lipid Mediat. (2020) 146:106405. 10.1016/j.prostaglandins.2019.106405 31838196

[34] Shoieb SM El-Sherbeni AA El-Kadi AOS. Subterminal hydroxyeicosatetraenoic acids: crucial lipid mediators in normal physiology and disease states. Chem Biol Interact. (2019) 299:140–50. 10.1016/j.cbi.2018.12.004 30543782

[35] Xu X Li R Chen G Hoopes SL Zeldin DC Wang DW. The role of cytochrome P450 epoxygenases, soluble epoxide hydrolase, and epoxyeicosatrienoic acids in metabolic diseases. Adv Nutr. (2016) 7:1122–8. 10.3945/an.116.012245 28140329 PMC5105036

[36] Dos Santos LRB Fleming I. Role of cytochrome P450-derived, polyunsaturated fatty acid mediators in diabetes and the metabolic syndrome. Prostaglandins Other Lipid Mediat. (2019) 148:106407. 10.1016/j.prostaglandins.2019.106407 31899373

[37] Valdes AM Ravipati S Pousinis P Menni C Mangino M Abhishek A. Omega-6 oxylipins generated by soluble epoxide hydrolase are associated with knee osteoarthritis. J Lipid Res. (2018) 59:1763–70. 10.1194/jlr.P085118 29986999 PMC6121933

[38] Morisseau C Hammock BD. Impact of soluble epoxide hydrolase and epoxyeicosanoids on human health. Annu Rev Pharmacol Toxicol. (2013) 53:37–58. 10.1146/annurev-pharmtox-011112-140244 23020295 PMC3578707

[39] Zhang H Falck JR Roman RJ Harder DR Koehler RC Yang ZJ. Upregulation of 20-HETE synthetic cytochrome P450 isoforms by oxygen-glucose deprivation in cortical neurons. Cell Mol Neurobiol. (2017) 37:1279–86. 10.1007/s10571-017-0462-8 28110484 PMC5522363

[40] Knudsen JG Bertholdt L Gudiksen A Gerbal-Chaloin S Rasmussen MK. Skeletal muscle interleukin-6 regulates hepatic cytochrome P450 expression: effects of 16-week high-fat diet and exercise. Toxicol Sci. (2018) 162:309–17. 10.1093/toxsci/kfx258 29177473

[41] Rasmussen MK Bertholdt L Gudiksen A Pilegaard H Knudsen JG. Impact of fasting followed by short-term exposure to interleukin-6 on cytochrome P450 mRNA in mice. Toxicol Lett. (2018) 282:93–9. 10.1016/j.toxlet.2017.10.011 29030272

[42] Zhang Q Wang X Yan G Lei J Zhou Y Wu L. Anti- versus pro-inflammatory metabololipidome upon cupping treatment. Cell Physiol Biochem. (2018) 45:1377–89. 10.1159/000487563 29462800

[43] Heemskerk MM Dharuri HK van den Berg SA Jónasdóttir HS Kloos DP Giera M. Prolonged niacin treatment leads to increased adipose tissue PUFA synthesis and anti-inflammatory lipid and oxylipin plasma profile. J Lipid Res. (2014) 55:2532–40. 10.1194/jlr.M051938 25320342 PMC4242446

[44] Tang JS Bozonet SM McKenzie JL Anderson RF Melton LD Vissers MCM. Physiological concentrations of blueberry-derived phenolic acids reduce monocyte adhesion to human endothelial cells. Mol Nutr Food Res. (2019) 63:1900478. 10.1002/mnfr.201900478 31216087

[45] Tang JS Vissers MCM Anderson RF Sreebhavan S Bozonet SM Scheepens A bioavailable blueberry-derived phenolic acids at physiological concentrations enhance nrf2-regulated antioxidant responses in human vascular endothelial cells. Mol Nutr Food Res. (2018) 62:1700647 10.1002/mnfr.201700647 29278300

[46] Akerstrom TC Fischer CP Plomgaard P Thomsen C van Hall G Pedersen BK. Glucose ingestion during endurance training does not alter adaptation. J Appl Physiol. (2009) 106:1771–79. 10.1152/japplphysiol.91534.2008 19228984

[47] Impey SG Hearris MA Hammond KM Bartlett JD Louis J Close GL. Fuel for the work required: a theoretical framework for carbohydrate periodization and the glycogen threshold hypothesis. Sports Med. (2018) 48:1031–48. 10.1007/s40279-018-0867-7 29453741 PMC5889771

[48] Merry TL Ristow M. Do antioxidant supplements interfere with skeletal muscle adaptation to exercise training? J Physiol. (2016) 594:5135–47. 10.1113/JP270654 26638792 PMC5023714

